# Evaluation of the first nutritional psychiatry and psychosomatics outpatient clinic: Protocol for a prospective study of an individualized biopsychosocial therapy approach

**DOI:** 10.1371/journal.pone.0339862

**Published:** 2026-01-05

**Authors:** Sabrina Mörkl, Anna Ramirez-Obermayer, Birgit DelFabro, Sonja Lackner, Sandra Holasek, Julia Traub, Katharina Größbacher, Andreas Brandstätter, Marilena Wilding, Jolana Wagner-Skacel

**Affiliations:** 1 Department for Medical Psychology, Psychosomatics and Psychotherapy, Medical University of Graz, Graz, Steiermark, Austria; 2 Division of Immunology, Medical University of Graz, Graz, Steiermark, Austria; 3 Department of Internal Medicine, Joint Facilities, Medical University of Graz, Graz, Steiermark, Austria; Medical University of Vienna, AUSTRIA

## Abstract

**Background:**

Psychiatric and psychosomatic conditions are prevalent, disabling, and frequently resistant to standard treatments. Nutrition-focused care represents an emerging integrative approach, but clinical evidence remains scarce. The outpatient department for Nutritional Psychiatry and Nutritional Psychosomatics (NP) at the Medical University of Graz was established as a pilot project, offering add-on therapy with targeted nutritional counseling, nutrient substitution and supplementation, and interventions aiming to improve the gut–brain axis.

**Methods:**

This prospective, monocentric, observational study will include approximately 84 new patients aged 18–65 over a 12-month period. Participants will complete online questionnaires at baseline and every 3 months, while routine blood samples will assess inflammatory and metabolic markers. The primary outcome is change in perceived stress, measured by the Perceived Stress Scale (PSS-10). Secondary outcomes include resilience, quality of life (SF-12, EQ-5D-5L), somatic symptoms (SSS-8), dietary habits (REAP-S v.2), psychodynamic structure (OPD-SFK), patient satisfaction, health literacy, and biomarker changes.

**Discussion:**

This study will provide the first systematic evaluation of a specialized outpatient clinic for Nutritional Psychiatry and Psychosomatics in Europe. Findings will inform the potential of nutrition-focused interventions as part of routine psychiatric and psychosomatic care and may contribute to the development of personalized, integrative treatment strategies.

**Trial registration:**

ClinicalTrials.gov NCT07050342

This study was prospectively registered at ClinicalTrials.gov (Identifier: NCT07050342), register date: 2025-06-24.

## Introduction

Psychiatric and psychosomatic disorders are among the most prevalent and disabling health conditions worldwide [1]. Despite advances in treatment, 10–30% of patients with depression do not respond adequately to standard therapies, such as psychotherapy or medication, and often continue to experience debilitating symptoms including fatigue, sleep problems, and cognitive impairment [2]. The global prevalence of psychiatric disorders rose substantially following public health crises such as the COVID-19 pandemic, when rates of anxiety and depression increased by around 25%, with pooled prevalences of 30% and 28%, respectively, across general populations [3]. High non-response rates, combined with increasing incidence, highlight the urgent need for innovative, integrative approaches that address the biopsychosocial dimensions of mental health [4].

Nutritional psychiatry is an emerging interdisciplinary field that investigates how diet and nutrients influence mental health through mechanisms such as systemic inflammation, oxidative stress, and gut–brain axis interactions [5–7]. Nutritional quality shapes mood, stress response, microbiome composition, and neurotransmitter metabolism, whereas disrupted dietary patterns can impair metabolism, reduce serotonin synthesis, and promote low-grade inflammation- factors implicated in anxiety and depression [8]. Epidemiological studies consistently support a protective role of healthy dietary patterns. For example, higher fruit and vegetable intake was linked to reduced distress and mood/anxiety disorders in a Canadian cohort [9]. Evidence from large-scale NHANES analyses further confirmed that dietary quality is inversely associated with depression, with several indices-HEI-2020, AHEI, and the Mediterranean Diet Index-demonstrating strong protective effects. The modified Planetary Health Diet Index emphasizing fruit intake (PHDI-Fruits) showed the most pronounced benefit [10] and higher overall adherence to the PHDI was associated with significantly reduced depression risk, with vitamin C, fiber, and selenium identified as key protective nutrients [11].

Mechanistic studies provide a biological rationale for these findings. Antioxidant properties, anti-inflammatory effects, and functional modulation of cellular processes explain how dietary patterns and nutraceuticals may impact mental health. Nutraceuticals, dietary extracts or supplements with validated health benefits, include both essential nutrients (e.g., fatty acids, proteins, vitamins, minerals) and non-essential bioactive compounds (e.g., polyphenols, flavonoids, carotenoids) [12]. They influence antioxidant defense, gene expression, mitochondrial integrity, and immune function, and have demonstrated protective effects across metabolic, cardiovascular, gastrointestinal, and neurodegenerative diseases [13]. Increasing evidence suggests that higher intake of fiber, phytochemicals, and omega-3 fatty acids is associated with better mental health outcomes, while deficiencies in amino acids, vitamins, minerals, fiber, and omega-3 fatty acids have been linked to distinct psychiatric conditions [5]. Randomized trials, such as the SMILES study, have shown that Mediterranean-style dietary interventions can significantly reduce depressive symptoms [5]. Preliminary evidence from a small Austrian field study suggests that individualized micronutrient supplementation, combined with lifestyle changes, can substantially improve depressive symptoms, highlighting the need for systematic screening of nutritional deficiencies in psychiatric care [14].

Despite this growing evidence, clinical implementation remains limited. Our international survey of over 1,000 mental health professionals across 52 countries revealed that although poor diet quality is widely recognized in psychiatric populations, only 0.8% rated their nutrition training as “very good” [15]. Nutritional aspects are also insufficiently represented in psychiatric guidelines, often limited to basic and non-specific recommendations [16–19]. This reflects a broader conceptual gap: while psychiatry traditionally refers to a biopsychosocial model, in practice it often narrows to a *pharmaco-psychosocial* approach, where the “biological” dimension is largely reduced to pharmacology. Broader biological factors, such as metabolism, inflammation, and nutrition, remain underrepresented in textbooks, training and clinical care, despite their increasing relevance for patient outcomes [15, 20].

Recent advances in Nutritional Psychiatry emphasize the need for rigorous, personalized approaches that move beyond single-nutrient supplementation, incorporating biomarkers (e.g., inflammation, nutrient status, microbiome profiles) and exploring synergistic strategies, such as combining psychobiotics with nutraceuticals [6].

To address this gap, the outpatient department for Nutritional Psychiatry and Nutritional Psychosomatics (NP) at the Medical University of Graz was established as the first dedicated multidisciplinary clinic for Nutritional Psychiatry in Europe. This project offers NP add-on care for patients, including targeted nutritional counseling, nutrient substitution and supplementation, and gut–brain axis modulation, delivered within a coordinated, patient-centered model.

This study protocol aims to evaluate whether personalized, nutrition-focused outpatient care can meaningfully reduce perceived stress (primary endpoint: PSS-10) and improve resilience, quality of life, somatic symptoms, dietary habits, psychodynamic structure, patient satisfaction, health literacy, and inflammatory/metabolic biomarkers over 12 months in patients of the NP outpatient department. To our knowledge, little is known about patient-reported outcomes in nutrition-focused psychiatric care beyond initial engagement and satisfaction. This study will therefore prospectively assess the impact of the NP outpatient clinic on perceived stress, health-related quality of life, and healthcare quality, providing important insights into the potential of integrated nutritional care in routine psychiatric and psychosomatic practice.

## Materials and methods

The primary objective of this study is to evaluate the potential impact of care delivered by the outpatient department for Nutritional Psychiatry and Nutritional Psychosomatics (NP) at the Medical University of Graz on perceived stress among adults with psychiatric and psychosomatic disorders. Specifically, the study aims to determine whether personalized, nutrition-focused care over a 12-month period leads to a statistically significant reduction in perceived stress, as measured by the Perceived Stress Scale (PSS-10) [21].

In addition to this primary aim, the study has several secondary objectives:

- To assess changes in **psychological resilience**, using the Brief Resilience Scale (BRS-D) [22].
- To evaluate changes in **health-related quality of life**, through the SF-12 and EQ-5D-5L instruments [23, 24].
- To document **changes in somatic symptom burden**, based on the Somatic Symptom Scale (SSS-8) [25].
- To monitor **dietary habits and quality**, using the Rapid Eating Assessment – Short Version (REAP-S v.2) and Food Frequency Items [26].
- To explore shifts in **psychodynamic functioning**, utilizing the Operationalized Psychodynamic Diagnostics Structure Questionnaire (OPD-SFK) [27].
- To measure **subjective health literacy**, as assessed both by patients and physicians.
- To capture **patient satisfaction** with the NP clinic’s care delivery model.
- To track changes in a broad panel of **laboratory biomarkers**, including inflammatory markers (e.g., CRP, IL-6), metabolic parameters (e.g., lipid profile, glucose metabolism), and vitamin status (e.g., vitamin D, vitamin B12, ferritin).

Collectively, these objectives are designed to provide preliminary data on the feasibility, acceptability, and potential effectiveness of the NP clinic’s integrated care model in routine practice. The findings will serve as a foundation for future research into the role of nutrition-focused, multidisciplinary interventions in improving psychosomatic health outcomes.

### Study design

This study is designed as a prospective, monocentric, observational study conducted at the Department of Medical Psychology, Psychosomatics, and Psychotherapy at the Medical University of Graz, Austria. The study aims to systematically evaluate the outcomes of care delivered by Europe’s first outpatient clinic dedicated to Nutritional Psychiatry and Nutritional Psychosomatics (NP).

Participants will include adult patients referred to the NP outpatient clinic who meet predefined eligibility criteria. Recruitment occurs consecutively at the patients’ first visit to the clinic. Participation is voluntary, with informed consent obtained prior to any study procedures.

Eligible participants will be followed for 12 months, with assessments scheduled at baseline and every 3 months thereafter. The assessment protocol comprises both patient-reported outcome measures (PROMs) and routine clinical laboratory tests. All questionnaires are completed digitally via secure online links provided by the EvaSys platform (Evasys GmbH, 2025). Blood samples are collected as part of routine clinical care and analyzed for a comprehensive panel of health-related biomarkers.

The study is non-randomized and participants receive individualized, nutrition-focused care as part of routine clinical practice; no experimental interventions, blinding, or control groups are involved. The NP clinic’s care model integrates nutritional counseling, dietary education, personalized supplementation and substitution where indicated, and psychosocial support aimed at optimizing gut-brain axis function and reducing patient burden. The nature of this study is exploratory: to collect preliminary data on patient-reported and biological outcomes, and assess feasibility in routine clinical care.

**Fig 1:**
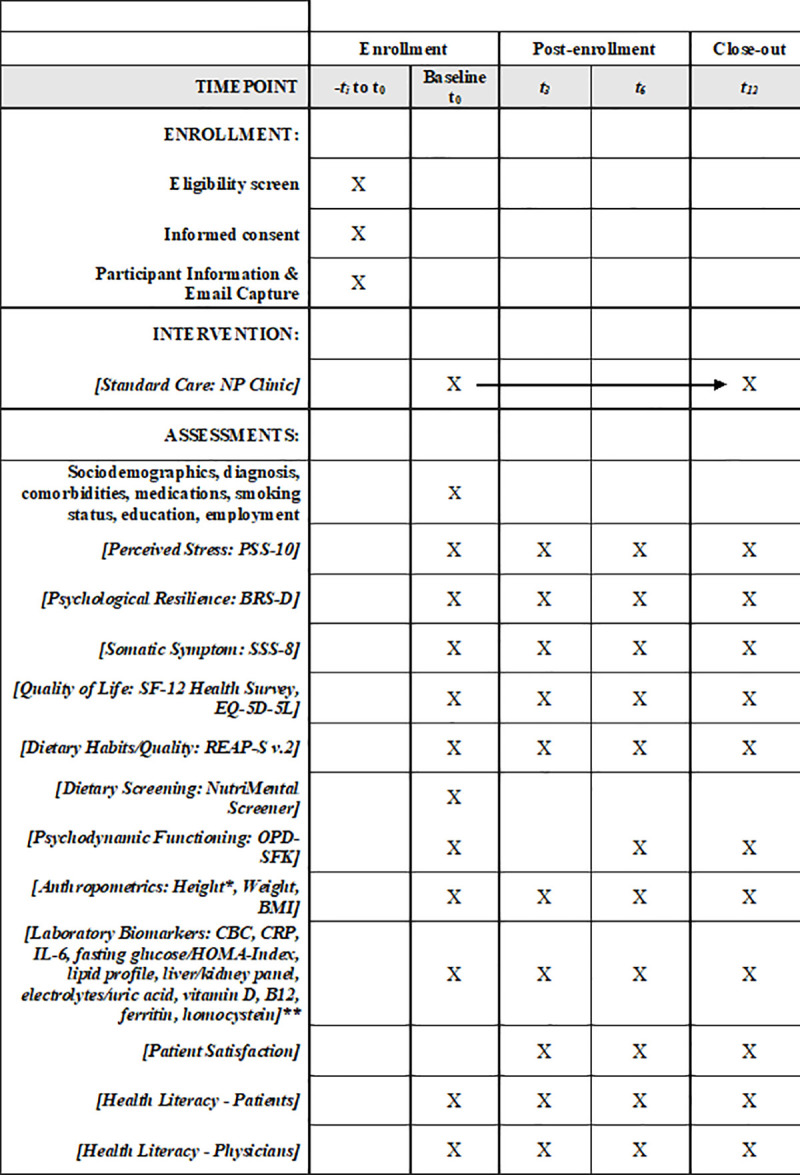
Participant flow diagram according to SPIRIT guidelines.

Fig 1 shows the participant flow diagram according to SPIRIT guidelines.

## Trial registration

### Patient involvement

The development of the research questions, outcome measures, and overall study design was informed by the study team’s extensive clinical interactions with patients during routine care. All participants will provide written informed consent prior to enrollment and will be thoroughly informed about the study’s aims, procedures, and potential risks and benefits. Throughout the study, patient feedback will be systematically collected using patient satisfaction questionnaires, which include both structured and open-ended items to capture participants’ perspectives on care quality and relevance. After study completion, participants will receive a summary of the study results, including key findings presented in an accessible format (e.g., newsletter), to acknowledge their contribution and promote knowledge translation. These patient perspectives will also inform the ongoing refinement of the care model and the design of future research on patient-centered, nutrition-focused psychosomatic care.

### Study setting

This is a prospective, monocentric, observational cohort study conducted at the NP outpatient clinic of the Medical University of Graz with the aim of evaluating patient-reported and biological outcomes of nutrition-focused care. The Outpatient Department for Nutritional Psychiatry and Nutritional Psychosomatics (NP) is part of the Department of Medical Psychology, Psychosomatics, and Psychotherapy at the Medical University of Graz and began operations in January 2024. The clinic is located at the University Hospital Graz and serves as a convenient and accessible point of care for patients referred from psychiatric, psychosomatic, and internal medicine services, as well as from primary care physicians. The NP clinic provides individualized, multidisciplinary care that includes nutritional counseling, education on gut-brain interactions, dietary and nutrient substitution and supplementation guidance, lifestyle advice, and psychosocial support. Care is coordinated by a multidisciplinary team comprising physicians, clinical psychologists, dietitians, and psychotherapists, ensuring integrated support for patients with complex psychiatric and psychosomatic conditions. All care is delivered as part of routine clinical practice, without experimental treatments.

### Timeline

This study has not yet started. Participant recruitment is planned to begin in spring 2026 and will continue until the target sample size is reached. Recruitment and data collection are both expected to be completed by June 1, 2028. The study results are anticipated by December 1, 2028. None of these stages have been completed yet.

### Participants/inclusion and exclusion criteria

Participants will be adults referred to the Nutritional Psychiatry (NP) outpatient clinic and other specialized psychosomatic outpatient services at the Medical University of Graz. Eligible participants must be new patients at the clinic, aged 18–65 years, with sufficient German language proficiency to provide informed consent and complete the assessments. Because all questionnaires are delivered electronically, participants must have access to email and be able to complete online forms.

### Inclusion criteria

1. Ability to provide written informed consent.
2. New patient at the NP outpatient clinic.
3. Age between 18 and 65 years.
4. Sufficient German language skills for participation and study procedures.
5. Access to email and the ability to complete online questionnaires.

### Exclusion criteria

1. Inability to provide informed consent or lack of consent.
2. Dementia or significant cognitive impairment, operationalized as a Mini-Mental State Examination (MMSE) score < 20.
3. Severe substance dependence (alcohol, benzodiazepines, opioids), based on clinical judgment at intake.
4. Severe physical, neurological, or motor impairments that would preclude participation in online assessments (e.g., inability to operate electronic devices).
5. No access to email or inability to use online questionnaires.
6. Active malignant disease.
7. Severe autoimmune disease or current immunosuppression, due to expected alterations in inflammatory readouts.

Participation is voluntary. Written informed consent is obtained before enrolment. All eligible patients attending their first clinic appointment will be invited to participate and will receive comprehensive study information beforehand.

### Interventions (care at the NP outpatients department)

All participants in this study will receive care as part of routine clinical services provided by the outpatient department for Nutritional Psychiatry (NP) at the Medical University of Graz. The NP clinic offers individualized, nutrition-focused, biopsychosocial care for patients with psychiatric and psychosomatic disorders, delivered by a multidisciplinary team consisting of physicians, clinical psychologists, dietitians, and psychotherapists. Care at the NP outpatient clinic encompasses nutritional counseling and education on the gut–brain axis, personalized dietary recommendations, and, where indicated, targeted nutrient substitution and supplementation [12, 14]. In addition, lifestyle counseling and psychosocial support are provided to promote psychological well-being and resilience.

### Interventions by discipline

#### Physicians (Psychosomatic Medicine/ Psychiatry).

Physicians conduct the comprehensive diagnostic assessment, including psychiatric, psychosomatic, and somatic evaluation; review laboratory and nutritional biomarkers; identify comorbidities; and develop the overall treatment plan. They provide medical management, including initiation or adjustment of psychopharmacological treatment when indicated, monitoring of physical health parameters, targeted nutrient substitution (e.g., vitamin D, B-vitamins, iron, omega-3 fatty acids) and nutritional counseling based on clinical need.

### Dietitians (Clinical Nutrition)

Dietitians deliver structured nutritional counseling tailored to psychiatric and psychosomatic conditions in individual and group settings. This includes dietary assessment, individualized meal planning, education on the gut–brain axis, food-mood interactions, anti-inflammatory dietary strategies, and guidance on nutrient-dense dietary patterns. They may also provide practical strategies for symptom-related eating difficulties (e.g., appetite changes, gastrointestinal symptoms).

### Clinical psychologists

Clinical psychologists provide psychoeducation, behavioral interventions, and lifestyle-oriented counseling related to stress, sleep, emotional eating, and coping strategies. Depending on patient needs, they may conduct brief cognitive-behavioral interventions, motivational interviewing, or stress-reduction techniques to support adherence to dietary and lifestyle recommendations.

### Psychotherapists

Psychotherapists offer evidence-based psychotherapeutic interventions targeted at underlying psychological patterns, interpersonal functioning, trauma-related symptoms, or chronic stress. Interventions may include cognitive-behavioral therapy, trauma-informed approaches, mindfulness-based interventions (such as mindful eating), or interpersonal strategies, depending on the therapist’s expertise and the patient’s clinical presentation.

### Nature of the intervention

Care is delivered as part of routine clinical practice, without experimental procedures, randomization, or blinding. All interventions are personalized, following a biopsychosocial and salutogenetic approach integrating mental, physical, and nutritional health. The focus is on enhancing resilience, improving psychological well-being, and addressing modifiable lifestyle and nutritional factors that influence psychiatric symptoms.

### Concomitant care

Permitted concomitant care includes all aspects of treatment-as-usual, such as ongoing psychopharmacological management by external physicians, existing psychotherapeutic treatments, or standard medical care. These treatments are documented but not restricted, as long as they form part of the participant’s established care.

Prohibited concomitant care includes any investigational treatments, experimental interventions, or simultaneous participation in another clinical trial during the study period, as these could confound outcome interpretation.

To minimize confounding, only standard medical and psychotherapeutic treatments that form part of the patient’s established routine care are permitted. These treatments are not expected to interfere with the study outcomes because (i) they reflect the usual clinical context in which the NP outpatient service operates, (ii) they are stable, ongoing components of care rather than newly initiated interventions during the study period, and (iii) the primary outcomes rely on within-person change over time. As a result, any influence of concurrent routine treatments would be consistent across the cohort and can be accounted for analytically through appropriate covariates and documentation.

### Outcome measures

#### Primary outcome.

The primary outcome of this study is the change in perceived stress levels over a 12-month period, as measured by the Perceived Stress Scale (PSS-10) [21]. The PSS-10 is a widely used, validated 10-item self-report questionnaire that assesses the degree to which individuals appraise their lives as stressful during the past month. Scores range from 0 to 40, with higher scores indicating greater perceived stress. Participants will complete the PSS-10 at baseline and at 3-month intervals throughout the study period. The primary hypothesis is that participants receiving care at the NP outpatient department will exhibit a statistically significant reduction in perceived stress scores after 12 months of individualized, nutrition-focused, psychosomatic care.

### Secondary outcomes

#### Patient health and care experience/ care quality.

Secondary outcomes include a comprehensive set of patient-reported and objective health outcomes, along with measures of patient experience and care quality, collected at baseline (T0) and at 3, 6, and 12 months (T3, T6, T12). Covariates will include relevant demographic and clinical characteristics.

### Patient health

- **Psychological resilience**

Measured via the Brief Resilience Scale (BRS-D) [22] at all time points.

- **Somatic symptom burden**

Assessed with the **Somatic Symptom Scale-8 (SSS-8)** [25] at all time points.

- **Quality of life**

Captured with two validated instruments: SF-12 Health Survey [23] and EQ-5D-5L [24], at all time points.

- **Dietary habits and quality**

Evaluated using the Rapid Eating Assessment for Participants – Short Version (REAP-S v.2) [26] at all time points.

- **Dietary baseline screening**

At baseline only, dietary risk factors will be assessed using the NutriMental Screener [28], providing a detailed profile of participants’ habitual diet and nutritional status.

- **Psychodynamic functioning**

Assessed using the Operationalized Psychodynamic Diagnostics – Structure Questionnaire (OPD-SFK) [27] at baseline, 6 months, and 12 months.

- **Physical health measures**

Height, weight, and body mass index (BMI) will be recorded at all clinical visits (T0, T3, T6, T12).

- **Biomarkers**

Laboratory biomarkers reflecting inflammation and metabolic status will be collected at all clinical visits (T0, T3, T6, T12), including:

◦ Differential blood count
◦ C-reactive protein (CRP)
◦ Interleukin-6 (IL-6)
◦ Fasting glucose and HOMA-Index
◦ Lipid profile (total cholesterol, HDL, LDL, triglycerides)
◦ Liver enzymes (ALT, AST, GGT)
◦ Kidney function markers (creatinine, urea)
◦ Electrolytes, uric acid
◦ Vitamin D, vitamin B12, ferritin, homocysteine

### Patient care experience/ care quality

- **Patient satisfaction**

Patient satisfaction will be measured at all follow-up time points using a questionnaire adapted from instruments applied in other outpatient services at the Medical University of Graz. The questionnaire covers organization and processes, competence and support from staff, communication, and overall patient impressions, with open-ended questions for qualitative feedback.

- **Health literacy**

Subjective health literacy will be assessed at all time points on a 10-point Likert scale by both participants and treating physicians. The physician questionnaire for this purpose will be completed separately at T0, T3, T6, and T12.

### Covariates: individual differences

Covariates collected at baseline include:

- Age
- Sex
- Educational attainment
- Employment status
- Smoking status
- Psychiatric diagnosis
- Chronic illnesses
- Current medication use
- Body mass index (BMI)

Fig 1 provides an overview of the measurement schedule across baseline and follow-up time points according to SPIRIT guidelines.

### Participant timeline: Schedule of enrollment, interventions, and assessments

**Fig 1** SPIRIT 2025 diagram of the schedule of enrolment, interventions, and assessments. BMI = Body Mass Index; BRS-D = Brief Resilience Scale – German version; CBC = Complete Blood Count; CRP = C-Reactive Protein; EQ-5D-5L = EuroQol 5-Dimensions, 5-Level version; HOMA-Index = Homeostatic Model Assessment Index; IL-6 = Interleukin-6; OPD-SFK = Operationalized Psychodynamic Diagnostics – Structure Questionnaire (Kurzform); PSS-10 = Perceived Stress Scale – 10 Items; REAP-S v.2 = Rapid Eating Assessment for Participants – Short Version, Version 2; SF-12 = 12-Item Short Form Health Survey; SSS-8 = Somatic Symptom Scale – 8 Items.

* Height measured at baseline only unless clinically indicated; weight and BMI measured at every visit.

** Blood samples are drawn as part of routine care and analyzed at the institutional laboratory of the Medical University of Graz.

### Sample size

As this is an exploratory study, no formal sample size calculation was performed. Based on clinic records, approximately 10 new patients are expected per month, with a projected participation rate of around 70%. Over a 12-month period, this yields an estimated sample size of approximately 84 participants. The chosen sample size is primarily based on feasibility and the expected number of eligible patients.

As this is a pilot study, one of its key methodological functions is to obtain empirical estimates of the variance structure of the primary outcome, namely the change in PSS-10 scores over 12 months. The study will generate estimates of the standard deviation and variance of the PSS-10 change score, as well as the intra-individual correlation between baseline and follow-up measurements. These metrics will provide information about the dispersion and temporal stability of perceived stress in this clinical population. The resulting variance and correlation estimates will form the basis for data-driven, precision-oriented sample size calculations for a future fully powered trial using an appropriate repeated-measures design.

### Recruitment, enrolment and consent

Participants will be recruited consecutively at their first visit to the NP outpatient clinic at the specialized psychosomatic outpatient services at the Medical University of Graz. Eligible patients will be identified during routine intake procedures and informed about the study by clinical staff at the reception desk. Written study information and the informed consent form will be provided to patients in advance, allowing sufficient time for review before their initial clinical consultation. During the first visit, treating physicians will provide additional oral information, answer any questions, and obtain written informed consent from patients who agree to participate. Participation is entirely voluntary and does not affect access to standard care. Enrolled participants will be asked to provide an email address for secure communication and will be invited to complete the baseline survey within one week following their initial visit.

### Data collection methods

Data will be collected at baseline and at 3, 6, and 12 months through a combination of patient-reported questionnaires and routine clinical assessments. Self-report measures will be administered digitally via the secure EvaSys platform, with participants receiving email invitations containing unique, time-limited links for online completion. The baseline survey will be distributed within one week of enrolment, with follow-up surveys sent automatically at scheduled intervals. Clinical and demographic information (e.g., age, sex, BMI, psychiatric diagnoses, current medications, smoking status, education, employment) will be extracted from routine clinic records at baseline. Blood samples will be collected during regular clinic visits approximately every three months and analyzed for a comprehensive panel of biomarkers reflecting inflammation, metabolic health, and nutritional status.

All study data will be managed in accordance with national data protection laws and the European Union General Data Protection Regulation (GDPR). Patient-reported, clinical, and laboratory data will be pseudonymized using unique study identifiers and stored in a password-protected research database hosted on secure institutional servers at the Medical University of Graz, with regular automated backups. Access to the database will be restricted to authorized study personnel. Paper-based records, including signed informed consent forms, will be securely stored in locked cabinets within the department. Data will be periodically reviewed for accuracy, completeness, and consistency. Upon study completion, the final pseudonymized dataset will be archived securely in line with institutional policies and applicable regulations. All analyses will be conducted on aggregated data to protect participant confidentiality.

### Statistics

#### Descriptive statistics.

Descriptive statistics will be used to summarize baseline characteristics of the study sample, including demographic variables (e.g., age, sex, education, employment status), clinical characteristics (e.g., psychiatric diagnoses, comorbidities, current medications), and health-related variables (e.g., BMI, smoking status). For continuous variables, means and standard deviations (or medians and interquartile ranges, if data are non-normally distributed) will be reported. For categorical variables, absolute frequencies and percentages will be provided. Descriptive summaries will also be used to characterize the distribution of primary and secondary outcome measures at each time point. These descriptive analyses will facilitate the assessment of sample representativeness, inform interpretation of observed changes over time, and provide variance estimates for planning future studies.

### Primary outcome

The primary outcome – change in perceived stress over 12 months -will be analyzed using paired statistical methods to compare PSS-10 scores at baseline and at 12 months. Depending on data distribution, either a paired t-test (for normally distributed data) or a Wilcoxon signed-rank test (for non-normally distributed data) will be applied to assess the statistical significance of change. In addition, repeated-measures ANOVA or linear mixed-effects models will be employed to evaluate stress trajectories across all time points (baseline, 3, 6, and 12 months), allowing for the assessment of time effects and handling of missing data under the missing-at-random assumption. Model assumptions will be checked using residual plots and normality tests (e.g., Shapiro–Wilk). A two-sided *p*-value of <0.05 will be considered statistically significant. Effect sizes and 95% confidence intervals will be reported to provide an estimate of the magnitude and precision of observed changes.

### Secondary outcomes

Secondary outcomes will be analyzed using both cross-sectional and longitudinal statistical methods. For continuous variables such as resilience (BRS-D), somatic symptom burden (SSS-8), quality of life (SF-12, EQ-5D-5L), dietary quality (REAP-S v.2), psychodynamic functioning (OPD-SFK), patient satisfaction, subjective health literacy, and laboratory biomarkers, descriptive summaries (means, standard deviations, medians, interquartile ranges) will be provided at each time point. Changes over time will be evaluated using repeated-measures ANOVA or linear mixed-effects models, depending on the distribution and completeness of data, to account for within-subject correlations and potential missing data under the missing-at-random assumption.

Categorical outcomes and ordinal scales will be analyzed using non-parametric tests (e.g., Friedman test for repeated measures) or generalized estimating equations (GEE) where appropriate. Pairwise comparisons between baseline and each follow-up point (3, 6, 12 months) will include adjustment for multiple testing using the false discovery rate (FDR) methods.

Correlational analyses (Pearson or Spearman, depending on data distribution) will explore associations between changes in psychological outcomes and changes in biological markers over time. All tests will be two-sided, with statistical significance set at *p* < 0.05. Effect sizes (e.g., Cohen’s d, correlation coefficients) and 95% confidence intervals will be reported where appropriate to aid interpretation of clinical relevance.

### Missing data handling

All efforts will be made to minimize missing data, including the use of automated reminders for online questionnaires and coordination with scheduled clinic visits for follow-up assessments. Nevertheless, missing data are expected in a longitudinal study of this nature. The extent and patterns of missing data will be systematically assessed and reported [29].

For the primary outcome (PSS-10) and continuous secondary outcomes, if data are missing at random (MAR), analyses will be conducted using linear mixed-effects models, which provide unbiased estimates under the MAR assumption by using all available data points without requiring imputation. As a sensitivity analysis, multiple imputation by chained equations (MICE) will be applied to account for potential biases due to missing data. Imputed datasets will be pooled according to Rubin’s rules to obtain final estimates [30].

For categorical variables, appropriate methods such as GEE with robust standard errors will be employed, which accommodate incomplete repeated measures data [31]. Complete case analyses will be reported alongside imputation-based results for transparency. The reasons for missing data, if known (e.g., withdrawal, loss to follow-up), will be documented and included in the final report.

### Approach to multiple comparisons and statistical error management

To address the number of primary and secondary outcomes, the analysis will rely on multivariable mixed-effects models that account for within-subject correlation and thereby reduce inflation of Type I error inherent in multiple testing. Secondary outcomes that are not included in the primary mixed-model framework will be analyzed as exploratory, and their interpretation will explicitly consider the increased risk of false positives. Where appropriate, false discovery rate (FDR) control will be applied to secondary analyses to maintain reasonable Type I error control without compromising sensitivity in this pilot context.

### Monitoring

A formal Data Monitoring Committee (DMC) will not be established for this study. The project is a monocentric, non-interventional, prospective observational cohort evaluating routine care within the Nutritional Psychiatry and Psychosomatics Outpatient Clinic at the Medical University of Graz. As there is no randomization, investigational treatment, or high-risk intervention, the formation of an independent DMC is not required.

Data quality and participant safety will instead be ensured through the internal monitoring procedures of the study team and the institutional quality assurance structures of the Medical University of Graz. The study is conducted under ethics approval in compliance with the Declaration of Helsinki and institutional data protection policies.

Regular internal reviews of recruitment, data completeness, and adverse event documentation will be carried out by the principal investigator and co-investigators. Any protocol deviations or unexpected events will be documented and, if necessary, reported to the local ethics committee.

### Interim analysis

No interim analyses or formal stopping guidelines are planned. As this is a non-interventional, observational cohort study without randomization or investigational treatment, no early termination for efficacy or harm is anticipated.

Study progress and data quality will be reviewed periodically by the principal investigator and co-investigators to ensure adherence to the approved protocol and ethical standards. Only the principal investigator will have access to cumulative study data during the ongoing project. Any decision to modify or discontinue the study prematurely would be made in consultation with the institutional ethics committee of the Medical University of Graz, based on safety or feasibility considerations.

### Ethics and dissemination

This study will be conducted in accordance with the Declaration of Helsinki and all relevant national and institutional regulations. Ethical approval has been obtained from the Ethics Committee of the Medical University of Graz prior to the start of the study (EK-No:1300/2024, approval date: 24^th^ Jan 2025). All participants will provide written informed consent before enrolment and will be informed about the study’s purpose, procedures, potential risks and benefits, and their right to withdraw at any time without affecting their access to clinical care.

Potential risks include physical, psychological, or laboratory-based abnormalities. Harms are defined as any adverse medical occurrences or undesired effects experienced by participants during the course of the study, regardless of their suspected relation to the intervention. Harms will be systematically assessed at three-month intervals through standardized self-report questionnaires and structured clinical check-ups conducted by trained investigators. Any adverse events reported spontaneously by participants or observed by the study team between visits will also be documented. The frequency, severity, and potential relationship to the study intervention will be evaluated and recorded in accordance with Good Clinical Practice (GCP) guidelines.

Any important modifications to the study protocol – including changes to study design, eligibility criteria, outcomes, data collection methods, or ethical aspects will be documented in a revised version of the protocol. Each amendment will be submitted for review and approval by the Ethics Committee of the Medical University of Graz prior to implementation.

All relevant stakeholders, including the study team, institutional quality assurance officers, and collaborating departments, will be promptly informed of approved modifications. Updates will also be reflected in the trial registry entry and, where applicable, in subsequent publications or public data repositories to ensure transparency and compliance with Good Clinical Practice standards.

Data will be handled confidentially, stored securely, and analyzed in pseudonymized form to protect participant privacy. Only authorized study personnel will have access to personal data, and all procedures will comply with the European Union General Data Protection Regulation (GDPR).

Upon completion of the study, the findings will be disseminated through publication in peer-reviewed journals and presentations at national and international scientific conferences. Participants will receive a summary of the study results in accessible language (e.g., via newsletter) to acknowledge their contribution and promote knowledge translation. The study team will also explore opportunities to present results to stakeholders involved in mental health care, ensuring that insights from this evaluation can inform future service development.

### Data management

Access to the study data will be strictly controlled to ensure confidentiality and compliance with data protection regulations. Only authorized members of the study team at the Medical University of Graz will have access to the pseudonymized dataset for the purpose of data management, analysis, and reporting. Identifiable personal information (e.g., consent forms, contact details) will be stored separately from research data in secure, access-restricted locations and will not be shared with third parties.

To ensure data integrity and completeness, several routine quality control procedures will be implemented throughout the study. All electronic data will be entered directly into the secure study database with automated range checks, logic checks, and mandatory field validations to prevent entry of implausible or incomplete values. A subset of records will undergo double-entry verification to identify and correct potential discrepancies. Any inconsistencies flagged by the system will be addressed using predefined data query procedures, and corrections will be documented in the audit trail. These measures are consistent with standard quality assurance practices for observational clinical studies.

The final, pseudonymized dataset will be securely archived following study completion, in accordance with institutional policies and legal requirements. No individual participant data will be made publicly available. However, aggregated results and key findings will be disseminated via publications and presentations, ensuring that no individual participant can be identified. Requests for additional data access by external researchers will be considered on a case-by-case basis and require formal data-sharing agreements, ethics approval, and compliance with applicable data protection laws.

## Discussion

This study protocol describes the design and planned evaluation of a novel, individualized, nutrition-focused care model implemented at Europe’s first Outpatient Department for Nutritional Psychiatry and Nutritional Psychosomatics (NP). The aim of this prospective observational study is to generate preliminary evidence on the feasibility, acceptability, and potential effectiveness of this integrated biopsychosocial approach for patients with psychiatric and psychosomatic conditions.

The primary focus is on changes in perceived stress, a key transdiagnostic indicator of mental and physical health burden, while secondary outcomes include patient-reported resilience, quality of life, dietary habits, psychodynamic functioning, satisfaction with care, subjective health literacy, and biological markers of inflammation and metabolism. By combining self-report data with routinely collected clinical and laboratory parameters, this study takes a comprehensive perspective on patient outcomes, healthcare quality, and patient experience.

To date, most evidence linking diet and mental health derives from epidemiological studies and randomized controlled trials focused on single dietary interventions or populations with specific diagnoses (e.g., major depressive disorder).

To date, most evidence linking diet and mental health derives from epidemiological studies and randomized controlled trials focused on single dietary interventions or populations with specific diagnoses (e.g., major depressive disorder). However, this narrow approach does not fully reflect clinical reality, where most patients present with a plethora of overlapping complaints-not only depression or anxiety, but also somatic problems such as gastrointestinal or dermatological symptoms. Few studies have systematically evaluated the impact of multidisciplinary, nutrition-focused care as part of routine outpatient services – and none, to our knowledge, have done so in a psychosomatic outpatient setting targeting a broad patient population. This study addresses this gap and reflects the growing interest in integrated care models that embrace nutrition as a modifiable factor within the biopsychosocial framework of mental healthcare.

Strengths of this study include its pragmatic, real-world design, the integration of validated patient-reported outcome measures and objective biomarkers, and the ability to capture patient satisfaction and care quality. Moreover, the study’s repeated-measures design enables the exploration of changes over time, providing important variance estimates and insights for planning future controlled trials.

However, several limitations should be acknowledged. The study is observational and non-randomized, so causal inferences regarding the effects of care at the NP clinic will be limited. Without a control group, it is not possible to rule out confounding influences such as regression to the mean, spontaneous remission, or concurrent treatments. The reliance on self-reported data for many outcomes introduces potential bias (e.g., recall or social desirability bias). The relatively small sample size will limit statistical power and precision of estimates, but the findings will nevertheless offer valuable insights for hypothesis generation and future study planning.

### Expected outcomes and clinical relevance

Based on prior work in nutritional psychiatry, lifestyle medicine, and gut–brain axis research, we anticipate that patients receiving multidisciplinary, nutrition-focused biopsychosocial care will show improvements across several domains. These may include reductions in depressive and anxiety symptoms, perceived stress, sleep disturbances, and fatigue, alongside improvements in mental well-being, quality of life, and eating-related behaviors. Given the close interplay between nutrition, inflammation, stress physiology, and gastrointestinal functioning, it is also expected that targeted nutritional and lifestyle interventions may be associated with reductions in gastrointestinal symptoms and greater self-efficacy in managing daily health challenges. In addition, we anticipate high patient satisfaction, reflecting the patient-centered and integrative design of the service.

In summary, this study will contribute new knowledge on patient-centered outcomes, healthcare quality, and the potential role of personalized nutrition-focused care in routine outpatient psychosomatic and psychiatric services. The results will inform future research, including the design of larger, controlled trials to rigorously test the efficacy and cost-effectiveness of this innovative care model.

## Supporting information

S1 Spirit Checklist Completed SPIRIT (Standard Protocol Items: Recommendations for Interventional Trials) 2013 checklist for the present study protocol, providing detailed information on all recommended items for clinical trial protocols. (DOCX)

S1 File Study Protocol German. (PDF)

S2 File Study Protocol English. (PDF)

## Abbreviations

ALT: Alanine Aminotransferase
AST: Aspartate Aminotransferase
BMI: Body Mass Index
BRS-D: Brief Resilience Scale (German version)
CRP: C-Reactive Protein
EQ-5D-5L: EuroQol 5-Dimensions, 5-Level Version
FDR: False Discovery Rate
GEE: Generalized Estimating Equations
GGT: Gamma-Glutamyltransferase
GDPR: General Data Protection Regulation
HDL: High-Density Lipoprotein
HOMA-Index: Homeostatic Model Assessment Index
IL-6: Interleukin-6
LDL: Low-Density Lipoprotein
MAR: Missing At Random
MICE: Multiple Imputation by Chained Equations
NP: Outpatient Department for Nutritional Psychiatry and Nutritional Psychosomatics
OPD-SFK: Operationalized Psychodynamic Diagnostics – Structure Questionnaire (Kurzform)
PSS-10: Perceived Stress Scale – 10 Items
REAP-S v.2: Rapid Eating Assessment for Participants – Short Version, Version 2
SF-12: 12-Item Short Form Health Survey
SSS-8: Somatic Symptom Scale – 8 Items
