# Supplementary material for: Evaluation of the first nutritional psychiatry and psychosomatics outpatient clinic: Protocol for a prospective study of an individualized biopsychosocial therapy approach
Source: PLoS One. 2026 Jan 5;21(1):e0339862. doi: 10.1371/journal.pone.0339862 (PMC12768340; doi:10.1371/journal.pone.0339862)
Supplement: S1 File — (PDF) [file pone.0339862.s002.pdf]

<u>Studienprotokoll – Version 3 vom 23.06.2025</u>

Evaluation der Spezialambulanzen der Abteilung für Medizinische Psychologie, Psychosomatik und Psychotherapie: eine Pilotstudie

Antragssteller

- Sabrina Leal Garcia
- Jolana Wagner-Skacel

Klin. Abteilung für Med. Psychologie, Psychosomatik und Psychotherapie Medical University of Graz (MUG), Auenbruggerplatz 3, A-8036 Graz
Phone (office): 00043/316/385/31520, Email: sabrina.moekrl@medunigraz.at

Forschungsteam

Dr. Anna Ramirez Obermayer
Dr. Birgit DelFabro
Dr. Rene Pilz
Cand. Med. Katharina Großbächer

Einleitung:
Diese prospektive Pilotstudie untersucht die Wirksamkeit der Betreuung und die Patientenzufriedenheit in Europas erster Spezialambulanz, die den Einfluss von Ernährung auf psychische und psychosomatische Gesundheit in den Fokus stellt. Darüber hinaus werden weitere Spezialambulanzen unserer Abteilung, insbesondere die Ambulanz für Psychokardiologie und Psychohepatologie evaluiert. Ziel der Studie ist es, Veränderungen im subjektiven Stressempfinden und in der psychischen Gesundheit der PatientInnen vor und während der Betreuung über einen Zeitraum von einem Jahr zu dokumentieren und zu bewerten. Darüber hinaus sollen mögliche Zusammenhänge zwischen psychischem Wohlbefinden und spezifischen ernährungsbedingten sowie physiologischen Parametern untersucht werden.

Methoden:
Es werden standardisierte psychologische Fragebögen eingesetzt, um Stressniveau, Resilienz, somatische Symptome und Lebensqualität der Teilnehmenden zu erfassen. Die Patientenzufriedenheit wird ebenfalls durch angepasste Fragen erhoben, die regelmäßig über den Studienzeitraum hinweg beantwortet werden. Zur Ermittlung möglicher physiologischer Korrelate werden Blutproben im Rahmen eines Routinelabors entnommen und auf relevante Marker wie Entzündungsfaktoren (z. B. CRP) analysiert. Die Erhebung erfolgt an der Baseline und in dreimonatigen Intervallen über den Studienzeitraum hinweg.

Erwartete Ergebnisse:
Es wird eine Reduktion des subjektiven Stressempfindens und eine Verbesserung des psychischen Wohlbefindens sowie der Lebensqualität erwartet. Auch wird mit einer positiven Veränderung der physiologischen Parameter, insbesondere der Entzündungsmarker, gerechnet, die eine unterstützende Wirkung der ernährungsbasierten Interventionen auf die psychosomatische Gesundheit nahelegt.

Diskussion:
Die Ergebnisse dieser Pilotstudie sollen Aufschluss über die Wirksamkeit ernährungsbasierter und allgemeiner Interventionen in der Psychosomatik geben und mögliche Optimierungspotenziale in der Patientenversorgung aufzeigen. Die Studie wird als Grundlage für weitere Forschungsarbeiten dienen und könnte dazu beitragen, das Konzept der Nutritional Psychiatry und der Ernährungspsychosomatik als innovative Behandlungsansätze in Europa weiter zu etablieren.

<u>Inhaltsverzeichnis</u>

1. Wissenschaftlicher Hintergrund
2. Ziele und Hypothesen
3. Studiendesign
4. Ein- und Ausschlusskriterien
5. Zielgrößen
    a. Hauptzielgrößen
    b. Nebenzielgrößen
6. Methoden
7. Statistik
8. Nutzen-Risiko-Analyse
9. Publikationen / Veröffentlichung der Ergebnisse
10. Zusammenfassung
11. Referenzen

Wissenschaftlicher Hintergrund

Psychische Erkrankungen und psychosomatische Beschwerden gehören weltweit zu den häufigsten und belastenden Gesundheitsproblemen. Die klassischen Therapiemethoden – bestehend aus Psychotherapie und medikamentöser Behandlung – sind jedoch in vielen Fällen weder kurativ noch präventiv ausreichend. Trotz intensiver Interventionen sprechen mehr als 40 % der PatientInnen nicht ausreichend auf die Therapie an (Maes et al., 2009), und rund 20–30 % bleiben therapieresistent und leiden weiterhin unter belastenden Symptomen wie Schlafstörungen, chronischen Schmerzen und Erschöpfung (Rush et al., 2006). Hinzu kommt eine steigende Inzidenz solcher Erkrankungen (GBD, 2019). Diese Lücken im Therapieerfolg und die unzureichenden präventiven Möglichkeiten unterstreichen die Notwendigkeit neuer Behandlungsansätze, die biologische, psychologische und soziale Faktoren im Sinne des bio-psycho-sozialen Modells stärker integrieren.

Ein innovativer Ansatz in der psychosomatischen Medizin und Psychiatrie ist die „Nutritional Psychiatry“ (Ernährungspsychiatrie). Diese aufstrebende Disziplin erforscht den Zusammenhang zwischen Ernährung, psychischer Gesundheit und körperlichen Symptomen und legt nahe, dass gezielte ernährungsbasierte Interventionen die mentale und physische Resilienz verbessern können. Forschungsergebnisse in der Ernährungspsychiatrie zeigen, dass die Wechselwirkungen zwischen Körper und Psyche stark durch Ernährung beeinflusst werden – insbesondere über die sogenannte Darm-Hirn-Achse. Diese bidirektionale Verbindung reguliert die Kommunikation zwischen dem zentralen Nervensystem und dem Darmmikrobiom und spielt eine zentrale Rolle bei der Steuerung von Stimmung und Stress (Mörkl et al., 2019).

Ein Ungleichgewicht im Darmmikrobiom, verursacht durch eine unausgewogene Ernährung, kann Entzündungen und Dysfunktionen bei der Neurotransmitterproduktion hervorrufen (Safadie et al., 2022). Diese Mechanismen tragen nachweislich zur Entstehung psychischer und psychosomatischer Erkrankungen bei, etwa durch die Beeinträchtigung des Serotoninhaushalts, dessen Hauptanteil (ca. 90 %) im Darm produziert wird und wesentlich die Stimmung beeinflusst. So wird beispielsweise der Serotoninvorläufer Tryptophan durch Entzündungsprozesse reduziert, wodurch die Serotoninproduktion beeinträchtigt wird. Studien zeigen, dass eine gezielte Ernährungsintervention das Gleichgewicht im Mikrobiom wiederherstellen und chronische Entzündungsprozesse reduzieren kann, die häufig mit Depressionen, Burnout und stressbedingten Erkrankungen assoziiert sind (Berding et al., 2023).

Eine entzündungshemmende Ernährungsweise, wie etwa die mediterrane Ernährungsweise, die reich an Omega-3-Fettsäuren, Antioxidantien und Ballaststoffen ist, hat sich als besonders vorteilhaft für die psychische Gesundheit erwiesen. Sie ist nicht nur mit einem geringeren Risiko für Depressionen verbunden, sondern wirkt präventiv auch gegen körperliche Symptome wie chronische Schmerzen und Entzündungen, die oft bei psychosomatischen Erkrankungen auftreten (Jacka et al., 2017). Diese Erkenntnisse deuten darauf hin, dass ein ernährungsbasiertes Interventionsmodell die psychosomatische Behandlung

erweitern könnte, indem es gezielt eine umfassendere Balance zwischen Körper und Psyche fördert und so Genesung und Resilienz unterstützt.

Vor diesem Hintergrund bietet die Spezialambulanz für Psychosomatik und Nutritional Psychosomatics am LKH Graz einen neuen Ansatz zur Integration individuell abgestimmter Interventionen, die sowohl die Ernährung als auch andere gesundheitsfördernde Faktoren berücksichtigen, in die psychiatrische und psychosomatische Behandlung. Die Ambulanz verfolgt ein personalisiertes, individuell auf die Bedürfnisse der PatientInnen zugeschnittenes Behandlungskonzept, das sich an der Salutogenese orientiert. Ziel ist es, nicht nur Symptome zu lindern, sondern auch die gesunden Anteile der PatientInnen zu fördern und ihre Resilienz zu stärken.

Diese prospektive Pilotstudie wird untersuchen, wie sich gezielte, personalisierte Ernährungsinterventionen auf psychische und körperliche Symptome – insbesondere das subjektive Stressempfinden – bei PatientInnen auswirken, die an psychischen und psychosomatischen Beschwerden leiden. Damit sollen neue Erkenntnisse zur praktischen Anwendung der Nutritional Psychiatry gesammelt und wichtige Grundlagen für zukünftige integrative Therapieformen geschaffen werden, die Körper und Geist gemeinsam ansprechen und eine nachhaltige Verbesserung der Lebensqualität und Gesundheit ermöglichen.

Im Rahmen der Klinischen Abteilung für Medizinische Psychologie, Psychosomatik und Psychotherapie am LKH-Univ. Klinikum Graz wurden weitere spezialisierte psychosomatische Ambulanzen etabliert, die den steigenden Bedarf an integrierter Versorgung in somatischen Fachbereichen adressieren: die Ambulanz für Psychokardiologie und jene für psychohepatologische und gastroenterologische Erkrankungen. Die **Psychokardiologie** widmet sich PatientInnen mit Herz-Kreislauf-Erkrankungen wie Myokardinfarkt, Herzinsuffizienz oder Herzrhythmusstörungen, die häufig von psychischen Belastungen wie Angststörungen, Depression oder Anpassungsschwierigkeiten begleitet werden. Auch psychosoziale Faktoren, wie chronischer Stress oder Lebensstilprobleme (z. B. Bewegungsmangel, inadäquate Ernährung), können zur Krankheitsentstehung beitragen oder den Verlauf negativ beeinflussen. In der Spezialambulanz, die in enger Kooperation mit der Abteilung für Kardiologie jeden Dienstag stattfindet, werden PatientInnen daher multidisziplinär im Rahmen von diagnostischen, psychotherapeutischen und edukativen Maßnahmen betreut. Die **Ambulanz für Psychohepatologie und Psychogastroenterologie** richtet sich an Menschen mit chronischen Magen-Darm- oder Lebererkrankungen, insbesondere solchen mit erhöhtem psychischen Belastungspotenzial – etwa aufgrund einer Alkoholabhängigkeit, einer chronisch-entzündlichen Darmerkrankung, nichtalkoholischer Fettleber oder Leberzirrhose. Psychische Komorbiditäten wie Depression, Angststörungen oder maladaptive Krankheitsverarbeitung sind in dieser Population häufig und mit reduzierter Adhärenz und Lebensqualität assoziiert. Das Behandlungsprogramm umfasst psychologische Diagnostik, Einzel- und Gruppentherapie, Lebensstilberatung und motivierende Gesprächsführung. Beide Ambulanzen basieren auf einem **Integrated Care-Modell**, das psychische, soziale und somatische Aspekte systematisch miteinander verknüpft und dadurch gezielt PatientInnen versorgt, deren psychosomatische Versorgung in der Regelversorgung oft unzureichend abgebildet ist. Die hohe Inanspruchnahme sowie die Rückmeldungen aus den kooperierenden klinischen Fachbereichen bestätigen den

Bedarf für diese spezialisierten, interdisziplinären Angebote. Ziel der vorliegenden Untersuchung ist es, neue Erkenntnisse zur praktischen Umsetzung integrierter psychosomatischer Versorgung in der Kardiologie sowie in der Gastroenterologie und Hepatologie zu gewinnen. Dabei sollen zentrale Grundlagen für zukünftige therapeutische Ansätze geschaffen werden, die psychische und körperliche Gesundheit systematisch gemeinsam adressieren. Durch die Evaluation dieser spezialisierten Ambulanzen soll ein Beitrag zur Entwicklung nachhaltiger, ganzheitlicher Behandlungsformen geleistet werden, die die Lebensqualität und das gesundheitliche Gesamtwohl der PatientInnen langfristig verbessern.

Ziele und Hypothesen

Die vorliegende Pilotstudie zielt darauf ab, eine erste Evaluation der Spezialambulanz für Ernährung und Psyche durchzuführen sowie weitere Spezialambulanzen mit innovativen Versorgungskonzepten unserer Abteilung zu evaluieren. Dabei sollen initiale Daten zu Behandlungserfolgen und zur Patientenzufriedenheit erfasst und analysiert werden. Die gewonnenen Ergebnisse dienen dazu, Optimierungspotenziale in der Patientenversorgung zu identifizieren und gezielt umzusetzen.

Die zentrale Forschungsfrage dieser Studie lautet: Kann durch die kontinuierliche Betreuung in Europas erster Spezialambulanz für Ernährung und Psyche nach einem Jahr Behandlungszeit eine statistisch signifikante Reduktion des Stresslevels bei PatientInnen mit psychosomatischen Erkrankungen erreicht werden? Ist ein Einfluss auf das Stresslevel auch in unsren anderen Spezialambulanzen zu verzeichnen?

Im Rahmen der individuell abgestimmten Interventionen, die auf Nährstoffversorgung, Ernährung, die Darm-Gehirn-Achse und Zuwendungsmedizin abzielen, erwarten wir einen signifikanten Rückgang der Stresssymptome der PatientInnen.

Hypothesen

Die Nullhypothese der Studie lautet: Es kommt zu keiner statistisch signifikanten Reduktion des Stresslevels bei PatientInnen mit psychosomatischen Erkrankungen nach einem Jahr Behandlungszeit in den Spezialambulanzen unserer Abteilung.

Die Alternativ Hypothese lautet: Nach einem Jahr Behandlungszeit in der Spezialambulanz für Ernährung und Psyche kommt es zu einer statistisch signifikanten Reduktion des Stresslevels bei PatientInnen mit psychosomatischen Erkrankungen in den Spezialambulanzen unserer Abteilung.

Studiendesign

Diese Studie wird an der **Klin. Abteilung für Med. Psychologie, Psychosomatik und Psychotherapie** als monozentrische, prospektive Pilotstudie durchgeführt.

PatientInnen werden durch niedergelassene PsychiaterInnen und/oder AllgemeinmedizinerInnen zugewiesen, ebenso über die KollegInnen der Inneren Medizin (Kardiologie/Gastroenterologie/Hepatologie). Die Rekrutierung der PatientInnen erfolgt im Rahmen des Erstgesprächs in der Spezialambulanz. Die PatientInnen werden bereits bei der Anmeldung am Ambulanzschalter über die Möglichkeit einer Teilnahme informiert und erhalten den Aufklärungsbogen, um diesen bereits vor dem Gespräch selbstständig durchlesen zu können. Während des Erstgespräches werden die PatientInnen nochmals von den ÄrztInnen umfassend

über die Studie aufgeklärt. Die Teilnahme erfolgt freiwillig und nach schriftlicher Einverständniserklärung.

Die TeilnehmerInnen werden Fragebögen zur Baseline und folglich in dreimonatigen Abständen für ein Jahr ausfüllen. Laboruntersuchungen werden im Rahmen regulärer Kontrolltermine in der Ambulanz, welche ebenfalls dreimonatlich stattfinden, durchgeführt. Die Fragebögen werden mittels des EvaSys-Systems online auszufüllen sein. Ein Link dazu wird den Teilnehmenden mittels E-Mails zukommen gelassen.

Ablauf Baseline
Nach dem Erstgespräch erhalten die TeilnehmerInnen eine E-Mail mit einem Link zu den Baseline Fragebögen, die sie binnen einer Woche ausfüllen sollen. Folgende Erhebungen werden durchgeführt:

- Einschätzung der Gesundheitskompetenz
- Baseline allgemeine Patientenzufriedenheit
- NutriMental Screener
- Perceived Stress Scale (PSS10)
- SF-12
- BRS-D (Resilienz)
- Somatic Symptom Scale
- EQ-5D-5L
- Food Frequency Items
- OPD-SFK

Ablauf Follow-Up
In dreimonatigen Abständen erhalten die TeilnehmerInnen erneut ein E-Mail mit einem Link zu folgenden Erhebungen:

- Einschätzung der Gesundheitskompetenz
- Follow-Up allgemeine Patientenzufriedenheit
- Perceived Stress Scale (PSS10)
- SF-12
- BRS-D (Resilienz)
- Somatic Symptom Scale
- EQ-5D-5L
- Food Frequency Items
- OPD-SFK

Ein-und Ausschlusskriterien

Die **Einschlusskriterien** sind: Informed Consent, NeupatientIn der Spezialambulanzen, Alter zwischen 18 und 65, Deutsche Sprache; Vorhandene Möglichkeiten und imstande sein, die Fragebögen online via E-Mail abzurufen und ausfüllen zu können.

Die **Ausschlusskriterien** sind: Mangelnde Einwilligung oder Einwilligungsfähigkeit, Demenz (Mini Mental Score <20), Ausgeprägte Abhängigkeit von Alkohol und/oder psychotroper Substanzen (Benzodiazepine, Morphine), schwere körperliche,

neurologische und motorische Einschränkungen, welche das Beantworten der Fragebögen unmöglich macht, Keine Möglichkeit oder Fähigkeit, die Fragebögen online abzurufen und auszufüllen, Tumorerkrankungen, Schwere Autoimmunerkrankungen oder Immunsuppression.

Zielgrößen

Hauptzielgrößen

- Zielparameter für die Haupthypothese ist das subjektive Stressempfinden (PSS-Score)

Nebenzielgrößen

- Scores der Fragebögen zu Resilienz, Lebensqualität, Somatische Symptome, Persönlichkeitsstruktur
- Ernährungsanamnese: Food Frequency Items
- Patientenzufriedenheit
- Subjektive Gesundheitskompetenz
- Blutparameter: Differential-Blutbild, CRP, IL-6, Nüchtern-BZ, HOMA-Index, Cholesterol, HDL, LDL, Triglyzeride, ALT, AST, GGT, Crea, Harnstoff, Elektrolyte, Harnsäure, Vitamin D, Ferritin, Homocystein, Vitamin B12
- Demographische Daten

Methoden

Intervention

Teilnehmende erhalten zur Baseline und folglich in dreimonatigen Abständen einen Link zu den Fragebögen (via EvaSys-System). Sie werden gebeten, diese binnen einer Woche auszufüllen. Standardlabore werden ebenfalls in dreimonatigen Abständen durchgeführt, allerdings im Rahmen der normalen Kontrolltermine in der Spezialambulanz. Eine rein studienbezogene Laborabnahme gibt es nicht.

Fragebögen

Die *Perceived Stress Scale 10 (PSS10)* wird zur Erhebung der subjektiven Stressbelastung verwendet.
Der *SF-12* ist ein Fragebogen zur Messung der gesundheitsbezogenen Lebensqualität. Es ist einer verkürzte Version des SF-36 und enthält Fragen zu körperliche Funktionen und Einschränkungen, Schmerzempfinden, Allgemeiner Gesundheitszustand, Vitalität, Soziale und emotionale Einschränkungen und Psychischen Belastung. Ergänzend wird die *EQ-5D-5L* erhoben, um ein umfassenderes Bild über die Lebensqualität zu erhalten.

Das Resilienz-Vermögen wird mittels *Brief Resilience Scale (BRS-D)* erfasst. Die *Somatic Symptom Scale (SSS*) gibt Informationen über erlebte somatische Symptome.

Zur Erfassung der psychischen Struktur wird die Kurzversion des *OPD-Strukturfragebogens (OPD-SFK)* eingesetzt. Der Fragebogen basiert auf dem Manual der Operationalisierten Psychodynamischen Diagnostik (OPD-2) und dient der Selbsteinschätzung zentraler struktureller Fähigkeiten, insbesondere in den Bereichen Selbst- und Fremdwahrnehmung, Steuerungsfähigkeit, emotionale Regulation und Beziehungsgestaltung. Die Kurzversion ermöglicht mit 12 Items eine ökonomische und zugleich valide Erhebung struktureller Funktionsniveaus im klinischen Kontext. Die Skalen zeigen eine gute interne Konsistenz sowie kriteriumsbezogene Validität in verschiedenen PatientInnen-Populationen. Ein stabiles Strukturniveau gilt als zentrale Voraussetzung für eine gesunde Stressverarbeitung, da es die Fähigkeit zur Selbstregulation in belastenden Situationen wesentlich beeinflusst.

Zur Baseline wird eine Ernährungsanamnese mittels *NutriMental Screener* durchgeführt, um einen Einblick in Ernährungsgewohnheiten und Ernährungsstatus zu bekommen.
Food Frequency Items werden sowohl zur Baseline, als auch bei den Follow-ups erfasst, um Ernährungsveränderungen und Compliance zu Ernährungsempfehlungen festzuhalten.

Um die Patientenzufriedenheit in der Spezialambulanz zu evaluieren, werden Fragebögen verwendet, die von denen anderer Ambulanzen des LKH Graz adaptiert worden sind. Dieser beinhaltet u.a. Fragen der Zufriedenheit hinsichtlich Organisation/Abläufe, Fachliche Kompetenz / Betreuung, Umgangston / Verhalten gegenüber PatientInnen, sowie offene Fragen über Gesamteindruck und Verbesserungsvorschläge.

Sowohl die PatientInnen als auch die behandelnden Ärztinnen werden angehalten, die Gesundheitskompetenz der PatientInnen auf einer Skala von 1 (sehr gering) – 10 (sehr hoch) zu allen Testzeitpunkten einzuschätzen.

Ergänzend erfassen wir folgende klinische und demographische Parameter: Alter, Geschlecht, Größe, BMI, Medikation, Rauchverhalten, chronische organische Erkrankungen, psychiatrische Diagnose, Bildungsstatus, Erwerbstätigkeit/Beschäftigungsausmaß.

Die Komplettierung der Baseline Fragebögen nimmt ca. 20 Minuten, die der Follow-Up Fragebögen ca 10-15 Minuten in Anspruch.

Standardlabor

Die Blutabnahmen werden zur Baseline und dann dreimonatig bei den normalen Kontrollterminen durch die MitarbeiterInnen in den Spezialambulanzen durchgeführt. Insgesamt werden ca. 45 ml Blut abgenommen (ca. 3 Esslöffel) und in einem Routine-Labor analysiert.

Folgende Routineparameter sind für diese Studie von besonderen Interesse: Differential-Blutbild, CRP, IL-6, Nüchtern-BZ, HOMA-Index, Cholesterol, HDL, LDL, Triglyzeride, ALT, AST, GGT, Crea, Harnstoff, Elektrolyte, Harnsäure, Vitamin D, Ferritin, Homocystein, Vitamin B12

Statistik

Datenauswertung und Evaluation

Die in dieser Studie erhobenen quantitativen Daten werden mit IBM SPSS analysiert. Zunächst erfolgt eine deskriptive Datenbeschreibung, bei der Mittelwert, Standardabweichung und Prozentränge berechnet werden, um die Grundcharakteristika der Stichprobe und der gemessenen Variablen darzustellen.

Um die Veränderung der Parameter im Verlauf der Behandlung zu analysieren, werden sowohl **verbundene t-Tests** als auch **Varianzanalysen mit Messwiederholung (ANOVA)** eingesetzt. Diese Verfahren ermöglichen es, Unterschiede zwischen den Messzeitpunkten (z. B. Baseline vs. Follow-up) zu bewerten und mögliche Effekte der Interventionen aufzuzeigen. Vor der Durchführung dieser Tests wird die Normalverteilung der Daten geprüft, beispielsweise durch den **Kolmogorov-Smirnov-Test** oder den **Shapiro-Wilk-Test**.

Zusätzlich werden die Zusammenhänge zwischen Variablen mittels Korrelationsanalysen untersucht. Abhängig von der Verteilung der Daten wird entweder der **Pearson-Korrelationskoeffizient** (für normalverteilte Daten) oder der **Spearman-Korrelationskoeffizient** (für nicht normalverteilte Daten) angewandt. Fehlerwahrscheinlichkeiten von $p < 0{,}05$ werden als statistisch signifikant bewertet.

Zur Untersuchung spezifischer Gruppenvergleiche und weiterer detaillierter Fragestellungen können zusätzlich post-hoc-Analysen durchgeführt werden.

Die qualitativen Daten aus den Fragebögen zur Patientenzufriedenheit werden durch Häufigkeitsanalysen und eine thematische Analyse ausgewertet, um häufige Themen und zentrale Aspekte der Zufriedenheit zu identifizieren. Diese ergänzen die quantitativen Ergebnisse und bieten einen umfassenderen Einblick in die Patientenperspektive.

Überlegung zur Fallzahl

Da es sich um eine Pilotstudie handelt, kann keine genaue Fallzahlplanung gegeben werden.
Von Jänner bis August 2024 verzeichnete die Spezialambulanz 91 Erstgespräche. Wenn man annimmt, dass die Zahl der NeupatientenInnen konstant bleibt, erwarten wir im Monat ca. 10 NeupatientenInnen d.h. 120 im Jahr. Wenn davon 70% an der Studie teilnehmen, kann die Zahl der StudienteilnehmerInnen grob auf ca. 84 Personen geschätzt werden.

Für die Psychokardiologie-Ambulanz wird mit einer regulären Frequenz von wöchentlich vier Terminen über 52 Kalenderwochen gerechnet, was einer

theoretischen Gesamtkapazität von 208 Terminen pro Jahr entspricht. Unter Berücksichtigung von Feiertagen, krankheitsbedingten Ausfällen und Urlaubszeiten ist realistischerweise mit einer Besetzung von etwa 75 % dieser Termine zu rechnen. Daraus ergibt sich eine geschätzte Zahl von ca. 150 verfügbaren Terminslots pro Jahr.

Da einzelne Patient:innen mehrfach vorstellig werden, liegt die tatsächliche Zahl einzelner NeupatientInnen unterhalb der Gesamtzahl an Kontakten. In vergleichbaren Versorgungssettings variiert die durchschnittliche Anzahl der jährlichen Kontakte pro PatientIn typischerweise zwischen 2,5 und 3 Terminen. Auf Basis dieser Erfahrungswerte ergibt sich ein realistisch geschätzter Umfang von rund 50 bis 60 Neukontakten pro Jahr.

In der Leberambulanz werden aktuell durchschnittlich 5–6 PatientInnen pro Montag betreut. Die tatsächliche Zahl variiert je nach Terminlage, Anwesenheit von StudienpatientInnen sowie kurzfristigen Absagen. Bei einer Schätzung von etwa 46 aktiven Ambulanztagen pro Jahr (unter Berücksichtigung von Feiertagen, Urlaub und Ausfällen) ergibt sich eine Jahresgesamtzahl von ca. 230 bis 276 PatientInnenkontakten.
Die Zahl der NeupatientInnen liegt schätzungsweise bei 1 bis maximal 2 pro Montag, was – bezogen auf 46 tatsächliche Ambulanztage – eine ungefähre Anzahl von 46 bis 92 Erstkontakten pro Jahr ergibt.

Ethik und Datenschutz

Die Studie wird gemäß den ethischen Richtlinien der Deklaration von Helsinki und den nationalen und internationalen Vorschriften durchgeführt. Die TeilnehmerInnen werden umfassend über die Ziele, Methoden, potenziellen Nutzen und Risiken der Studie aufgeklärt. Die Einwilligung zur Teilnahme erfolgt freiwillig und schriftlich. Die persönlichen Informationen der Teilnehmenden werden vertraulich behandelt und nur in anonymisierter Form in der Datenanalyse verwendet.

Nutzen Risiko Abwägung

Wir gehen davon aus, dass das subjektive Stressempfinden während der Betreuung in den Spezialambulanzen verringert werden kann. Durch das beantworten von Fragebögen sind kaum körperliche Beschwerden und/oder Begleiterscheinungen zu erwarten.

Durch die genaue und wiederholte Untersuchung biopsychosozialer Variablen (u.a. Stress, Ernährung, Resilienz, Lebensqualität), für die auch eine Nachbesprechung der Ergebnisse angeboten wird, erhalten die TeilnehmerInnen ebenfalls einen Einblick in mögliche persönliche Behandlungsfortschritte. Dieses Wissen können sie folglich dazu nutzen, sich gezielter mit Strategien zur Verbesserung des Lebensstils auseinanderzusetzen.

Das Auseinandersetzen mit den psychologischen Fragebögen könnte möglicherweise emotionale Reaktionen hervorrufen. Die ForscherInnen werden jedoch darauf achten, die Teilnehmenden vorab auf solche Möglichkeiten vorzubereiten und unterstützende

Maßnahmen anzubieten. Darüber hinaus könnte das Ausfüllen von Fragebögen auf dem Bildschirm zu Ermüdungserscheinungen und Kopfschmerzen führen. Es kann jedoch jederzeit eine Pause eingelegt werden und das Ausfüllen unter- oder abgebrochen werden. Die Routine-Blutabnahmen können vorübergehende Unannehmlichkeiten verursachen, wie leichte Schmerzen oder vorübergehende Blutergüsse an der Einstichstelle. In seltenen Fällen kann es zu Infektionen kommen. Die ForscherInnen werden sicherstellen, dass erfahrene Fachkräfte diese Prozeduren durchführen, um das Risiko von Komplikationen zu minimieren.

Die Studie wird gemäß den ethischen Richtlinien der Deklaration von Helsinki und den nationalen und internationalen Vorschriften durchgeführt. Die TeilnehmerInnen werden umfassend über die Ziele, Methoden, potenziellen Nutzen und Risiken der Studie aufgeklärt. Die Einwilligung zur Teilnahme erfolgt freiwillig und schriftlich. Die persönlichen Informationen der Teilnehmenden werden vertraulich behandelt und nur in anonymisierter Form in der Datenanalyse verwendet.

Durch diese Pilotstudie können wichtige Erkenntnisse über mögliche neue Behandlungs- und Präventionsmöglichkeiten in der Psychosomatik gesammelt werden. Zukünftig können weitere Studien auf Basis dieser durchgeführt werden und langfristig neue Therapie- und Präventionsmöglichkeiten entstehen.
Das Feedback der TeilnehmerInnen hinsichtlich ihrer Zufriedenheit in der Ambulanz ist von großer Bedeutung, um die Patientenversorgung zu optimieren.
Das Nutzen-Risiko Verhältnis ist deshalb positiv.

Rechtliche Aspekte

Die Studie wird im Einklang mit den geltenden nationalen und internationalen rechtlichen Bestimmungen sowie ethischen Richtlinien durchgeführt. Alle TeilnehmerInnen werden über ihre Rechte und Pflichten informiert und es wird sichergestellt, dass ihre Einwilligung freiwillig und informiert erfolgt. Die Datenschutzrichtlinien werden streng befolgt, um die Vertraulichkeit der erhobenen Daten zu gewährleisten.

Publikation und Veröffentlichung der Ergebnisse

Die Ergebnisse dieser Studie sollen in renommierten wissenschaftlichen Fachzeitschriften veröffentlicht werden, um die wissenschaftliche Gemeinschaft über die Erkenntnisse zu informieren. Autorenschaft und Veröffentlichungen erfolgen gemäß den Richtlinien der International Committee of Medical Journal Editors (ICMJE). Darüber hinaus werden die Ergebnisse auf wissenschaftlichen Konferenzen präsentiert, um den Austausch von Wissen und Erfahrungen mit anderen Fachleuten zu fördern.

Limitationen und Herausforderungen

Die vorliegende Pilotstudie weist einige Limitationen und Herausforderungen auf, die bei der Interpretation der Ergebnisse berücksichtigt werden müssen.

Erstens könnte die Studiendauer zu kurz sein, um statistisch signifikante Effekte oder nachhaltige Veränderungen zu beobachten, da sich die Wirkung von Ernährungs- und psychologischen Interventionen oft erst über einen längeren Zeitraum hinweg vollständig entfaltet. Zweitens ist die Studie auf eine kleine Teilnehmerzahl in der Spezialambulanz beschränkt, was die statistische Power und somit die Wahrscheinlichkeit, signifikante Ergebnisse zu finden, verringern kann. Dies erhöht das Risiko eines Typ-2-Fehlers, bei dem echte Effekte aufgrund unzureichender Power nicht als signifikant erkannt werden.

Ein weiteres potenzielles Problem ist der Einsatz von Selbstberichtsverfahren (Fragebögen), die anfällig für Verzerrungen wie soziale Erwünschtheit oder Erinnerungseffekte sind und damit die Reliabilität und Validität der erhobenen Daten beeinträchtigen können. Zudem ist die Compliance der TeilnehmerInnen hinsichtlich spezieller therapeutischer Empfehlungen in den Spezialambulanzen, wie zB. Ernährungsempfehlungen schwer kontrollierbar, und individuelle Unterschiede in der Nährstoffaufnahme und -verwertung könnten die Ergebnisse dieser Studie beeinflussen und zu einer erhöhten Variabilität führen, die unabhängig von der Intervention auftritt.

Aufgrund finanzieller und logistischer Einschränkungen ist es im Rahmen dieser Studie nicht möglich, spezifischere ernährungsdiagnostische Tests durchzuführen, die eine noch präzisere Anpassung der Interventionen ermöglichen würden. Diese Limitationen wirken sich auf die Aussagekraft und Verallgemeinerbarkeit der Ergebnisse aus, zeigen jedoch gleichzeitig wichtige Ansatzpunkte für zukünftige Studien auf. Diese könnten eine größere Stichprobe, längere Nachbeobachtungszeiträume und eine differenziertere ernährungsdiagnostische Vorgehensweise umfassen.

Zusammenfassung

Die vorliegende Pilotstudie zielt darauf ab, die Wirksamkeit einer Behandlung und die allgemeine Patientenzufriedenheit in Europas erster Spezialambulanz für Ernährung und Psyche sowie in den anderen Spezialambulanzen mit innovativen Versorgungskonzepten (Psychokardiologie/Psychohepatologie) zu untersuchen. Die Studie verwendet ein monozentrisches, prospektives Studiendesign und erfasst sowohl subjektive (Fragebögen) als auch objektive (Blutwerte) Parameter. Die Ergebnisse dieser Studie tragen dazu bei, die Patientenversorgung gezielt zu verbessern, und erste Daten hinsichtlich der Wirksamkeit klinisch bisher wenig erprobter ernährungsspezifischer Interventionen in der Psychosomatik zu sammeln.

Referenzen

Berding, K., Bastiaanssen, T.F.S., Moloney, G.M., Boscaini, S., Strain, C.R., Anesi, A., Long-Smith, C., Mattivi, F., Stanton, C., Clarke, G., Dinan, T.G., Cryan, J.F., 2023. Feed your microbes to deal with stress: a psychobiotic diet impacts microbial stability and perceived stress in a healthy adult population. Mol Psychiatry 28, 601-610.

GBD 2019 Mental Disorders Collaborators. Global, regional, and national burden of 12 mental disorders in 204 countries and territories, 1990–2019: a systematic analysis from the Global Burden of Disease Study 2019. *The Lancet Psychiatry*. 2022 Jan;9(1):30-49. doi: 10.1016/S2215-0366(21)00395-3.

Jacka FN, O'Neil A, Opie R, Itsiopoulos C, Cotton S, Mohebbi M, et al. A randomized controlled trial of dietary improvement for adults with major depression (the "SMILES" trial). *BMC Medicine*. 2017;15(1):23. doi:10.1186/s12916-017-0791-y.

Maes, M., Yirmyia, R., Noraberg, J., Brene, S., Hibbeln, J., Perini, G., Kubera, M., Bob, P., Lerer, B., Maj, M.J.M.b.d., 2009. The inflammatory & neurodegenerative (I&ND) hypothesis of depression: leads for future research and new drug developments in depression. 24, 27-53.

Mörkl S, Wagner-Skacel J, Lahousen T, Lackner S, Holasek SJ, Bengesser SA, Painold A, Holl AK, Reininghaus E. The Role of Nutrition and the Gut-Brain Axis in Psychiatry: A Review of the Literature. *Neuropsychobiology*. 2018 Sep 17:1-9. doi: 10.1159/000492834. Epub ahead of print. PMID: 30223263.

Rush AJ, Trivedi MH, Wisniewski SR, et al. Acute and longer-term outcomes in depressed outpatients requiring one or several treatment steps: a STAR*D report. *Am J Psychiatry*. 2006;163(11):1905–1917. doi:10.1176/ajp.2006.163.11.1905.

Safadi, J.M., Quinton, A.M.G., Lennox, B.R., Burnet, P.W.J., Minichino, A., 2022. Gut dysbiosis in severe mental illness and chronic fatigue: a novel trans-diagnostic construct? A systematic review and meta-analysis. Molecular Psychiatry 27, 141-153.
