# Supplementary material for: Evaluation of the first nutritional psychiatry and psychosomatics outpatient clinic: Protocol for a prospective study of an individualized biopsychosocial therapy approach
Source: PLoS One. 2026 Jan 5;21(1):e0339862. doi: 10.1371/journal.pone.0339862 (PMC12768340; doi:10.1371/journal.pone.0339862)
Supplement: S2 File — (PDF) [file pone.0339862.s003.pdf]

Study Protocol – Version 3 dated 23.06.2025

Evaluation of the Specialized Outpatient Clinics of the Department of Medical Psychology, Psychosomatics and Psychotherapy: A Pilot Study

Applicants
- Sabrina Leal Garcia
- Jolana Wagner-Skacel

Clinical Department of Medical Psychology, Psychosomatics and Psychotherapy, Medical University of Graz (MUG), Auenbruggerplatz 3, A-8036 Graz
Phone (office): 00043/316/385/31520, Email: sabrina.moerkl@medunigraz.at

Research Team

Dr. Anna Ramirez Obermayer
Dr. Birgit DelFabro
Dr. Rene Pilz
Cand. Med. Katharina Großbächer

Introduction:

This prospective pilot study examines the effectiveness of care and patient satisfaction in Europe's first specialized outpatient clinic that focuses on the influence of nutrition on mental and psychosomatic health. In addition, other specialized outpatient clinics of our department, in particular the clinic for psychocardiology and for psychohepatology, will be evaluated. The aim of the study is to document and assess changes in patients' subjective perception of stress and in their mental health before and during care over a period of one year. Furthermore, potential associations between psychological well-being and specific nutrition-related as well as physiological parameters will be investigated.

Methods:

Standardized psychological questionnaires will be used to assess participants' stress levels, resilience, somatic symptoms and quality of life. Patient satisfaction will also be recorded using adapted questions that are answered regularly over the course of the study period. To identify possible physiological correlates, blood samples will be taken as part of routine laboratory work and analyzed for relevant markers such as inflammatory factors (e.g., CRP). Data collection takes place at baseline and at three-month intervals throughout the study period.

Expected results:

A reduction in subjective stress perception and an improvement in psychological well-being as well as quality of life are expected. Positive changes in physiological parameters, particularly inflammatory markers, are also anticipated, suggesting a supportive effect of nutrition-based interventions on psychosomatic health.

Discussion:

The results of this pilot study are intended to provide insights into the effectiveness of nutrition-based and general interventions in psychosomatics and to highlight potential areas

for optimizing patient care. The study will serve as a basis for further research and could help to further establish the concept of Nutritional Psychiatry and Nutritional Psychosomatics as innovative treatment approaches in Europe.

Table of Contents

1. Scientific Background
2. Objectives and Hypotheses
3. Study Design
4. Inclusion and Exclusion Criteria
5. Outcome Measures
   a. Primary Outcome Measures
   b. Secondary Outcome Measures
6. Methods
7. Statistics
8. Benefit–Risk Analysis
9. Publications / Dissemination of Results
10. Summary
11. References

Scientific Background

Mental illnesses and psychosomatic complaints are among the most common and burdensome health problems worldwide. However, the classical therapeutic methods—consisting of psychotherapy and pharmacological treatment—are in many cases neither sufficiently curative nor preventive. Despite intensive interventions, more than 40% of patients do not respond adequately to therapy (Maes et al., 2009), and around 20–30% remain treatment-resistant and continue to suffer from distressing symptoms such as sleep disturbances, chronic pain and fatigue (Rush et al., 2006). In addition, there is a rising incidence of such disorders (GBD, 2019). These gaps in treatment success and the insufficient preventive options underline the need for new treatment approaches that more strongly integrate biological, psychological and social factors in the sense of the bio-psycho-social model.

An innovative approach in psychosomatic medicine and psychiatry is “Nutritional Psychiatry.” This emerging discipline explores the relationship between nutrition, mental health and physical symptoms and suggests that targeted nutrition-based interventions can improve mental and physical resilience. Research findings in nutritional psychiatry show that the interactions between body and psyche are strongly influenced by nutrition—particularly via the so-called gut-brain axis. This bidirectional connection regulates communication between the central nervous system and the gut microbiome and plays a central role in the regulation of mood and stress (Mörkl et al., 2019).

An imbalance in the gut microbiome, caused by an unbalanced diet, can trigger inflammation and dysfunctions in neurotransmitter production (Safadi et al., 2022). These mechanisms demonstrably contribute to the development of mental and psychosomatic disorders, for example by impairing the serotonin system, the majority of which (approx. 90%) is produced in the gut and substantially influences mood. For instance, the serotonin precursor tryptophan is reduced by inflammatory processes, thereby impairing serotonin production. Studies show that targeted dietary intervention can restore microbiome balance and reduce chronic inflammatory processes that are frequently associated with depression, burnout and stress-related disorders (Berding et al., 2023).

An anti-inflammatory dietary pattern, such as the Mediterranean diet, which is rich in omega-3 fatty acids, antioxidants and fiber, has proven particularly beneficial for mental health. It is not only associated with a lower risk of depression but also has preventive effects against

physical symptoms such as chronic pain and inflammation, which often occur in psychosomatic disorders (Jacka et al., 2017). These findings suggest that a nutrition-based intervention model could broaden psychosomatic treatment by specifically promoting a more comprehensive balance between body and mind, thereby supporting recovery and resilience.
Against this background, the Specialized Outpatient Clinic for Psychosomatics and Nutritional Psychosomatics at LKH Graz offers a new approach to integrating individually tailored interventions that consider both nutrition and other health-promoting factors into psychiatric and psychosomatic treatment. The clinic follows a personalized treatment concept tailored to the needs of the patients and oriented toward salutogenesis. The aim is not only to alleviate symptoms but also to promote patients' healthy capacities and strengthen their resilience.

This prospective pilot study will investigate how targeted, personalized dietary interventions affect mental and physical symptoms—particularly subjective stress perception—in patients suffering from mental and psychosomatic complaints. This will generate new insights into the practical application of Nutritional Psychiatry and establish important foundations for future integrative therapeutic approaches that address body and mind together and enable a sustainable improvement in quality of life and health.

Within the Clinical Department of Medical Psychology, Psychosomatics and Psychotherapy at the LKH University Hospital Graz, further specialized psychosomatic outpatient clinics have been established to address the growing need for integrated care in somatic specialties: the outpatient clinic for psychocardiology and the clinic for psychohepatological and gastroenterological diseases. Psychocardiology focuses on patients with cardiovascular diseases such as myocardial infarction, heart failure or cardiac arrhythmias, which are often accompanied by psychological burdens such as anxiety disorders, depression or adjustment difficulties. Psychosocial factors, such as chronic stress or lifestyle problems (e.g., lack of exercise, inadequate diet), can also contribute to disease development or negatively influence its course. In this specialized clinic, which takes place every Tuesday in close cooperation with the Department of Cardiology, patients receive multidisciplinary care in the context of diagnostic, psychotherapeutic and educational measures.

The clinic for psychohepatology and psychogastroenterology is aimed at people with chronic gastrointestinal or liver diseases, particularly those with increased psychological stress potential—for example due to alcohol dependence, inflammatory bowel disease, non-alcoholic fatty liver disease or liver cirrhosis. Psychological comorbidities such as depression, anxiety disorders or maladaptive illness processing are common in this population and are associated with reduced adherence and quality of life. The treatment program includes psychological diagnostics, individual and group therapy, lifestyle counseling and motivational interviewing. Both clinics are based on an integrated care model that systematically links psychological, social and somatic aspects and thereby specifically treats patients whose psychosomatic care is often inadequately represented in standard care. High utilization and feedback from the cooperating clinical departments confirm the need for these specialized, interdisciplinary services.

The aim of the present investigation is to gain new insights into the practical implementation of integrated psychosomatic care in cardiology as well as in gastroenterology and hepatology. The intention is to establish central foundations for future therapeutic approaches that systematically address mental and physical health together. By evaluating these specialized clinics, a contribution is to be made to the development of sustainable, holistic treatment forms that improve patients' quality of life and overall health in the long term.

Objectives and Hypotheses

This pilot study aims to conduct an initial evaluation of the Specialized Outpatient Clinic for Nutrition and Mental Health and to evaluate other specialized clinics with innovative care concepts within our department. Initial data on treatment outcomes and patient satisfaction will be collected and analyzed. The findings will be used to identify and implement targeted optimization potential in patient care.
The central research question of this study is: Can continuous care in Europe's first Specialized Outpatient Clinic for Nutrition and Mental Health achieve a statistically significant reduction in stress levels in patients with psychosomatic disorders after one year of treatment? Is there also an influence on stress levels in our other specialized outpatient clinics?
As part of the individually tailored interventions aimed at nutrient supply, nutrition, the gut-brain axis and relationship-based medicine, we expect a significant reduction in patients' stress symptoms.

Hypotheses

The null hypothesis of the study is: There is no statistically significant reduction in stress levels in patients with psychosomatic disorders after one year of treatment in the specialized outpatient clinics of our department.

The alternative hypothesis is: After one year of treatment in the Specialized Outpatient Clinic for Nutrition and Mental Health, there is a statistically significant reduction in stress levels in patients with psychosomatic disorders in the specialized outpatient clinics of our department.

Study Design

This study will be conducted at the Clinical Department of Medical Psychology, Psychosomatics and Psychotherapy as a single-center, prospective pilot study.

Patients will be referred by private psychiatrists and/or general practitioners, as well as by colleagues from internal medicine (cardiology/gastroenterology/hepatology). Recruitment takes place during the initial consultation in the specialized outpatient clinic. Patients are informed about the possibility of participation when registering at the clinic desk and receive the information sheet so that they can read it independently before the consultation. During the initial consultation, patients are again comprehensively informed about the study by the physicians. Participation is voluntary and requires written informed consent.

Participants will complete questionnaires at baseline and subsequently at three-month intervals for one year. Laboratory tests will be performed during regular follow-up appointments in the clinic, which also take place every three months. The questionnaires will be completed online using the EvaSys system. A link will be sent to participants by email.

Baseline Procedure

After the initial consultation, participants receive an email with a link to the baseline questionnaires, which they should complete within one week. The following assessments will be conducted:
• Assessment of health literacy

• Baseline general patient satisfaction
• NutriMental Screener
• Perceived Stress Scale (PSS10)
• SF-12
• BRS-D (resilience)
• Somatic Symptom Scale
• EQ-5D-5L
• Food Frequency Items
• OPD-SFK

Follow-Up Procedure
At three-month intervals, participants will again receive an email with a link to the following assessments:
• Assessment of health literacy
• Follow-up general patient satisfaction
• Perceived Stress Scale (PSS10)
• SF-12
• BRS-D (resilience)
• Somatic Symptom Scale
• EQ-5D-5L
• Food Frequency Items
• OPD-SFK

Inclusion and Exclusion Criteria

Inclusion criteria: Informed consent, new patient of the specialized outpatient clinics, age between 18 and 65, German language; having the means and being able to access and complete the questionnaires online via email.

Exclusion criteria: Lack of consent or capacity to consent, dementia (Mini-Mental Score < 20), pronounced dependence on alcohol and/or psychotropic substances (benzodiazepines, morphine), severe physical, neurological and motor impairments that make answering the questionnaires impossible, no possibility or ability to access and complete the questionnaires online, tumor diseases, severe autoimmune diseases or immunosuppression.

Outcome Measures

Primary Outcome Measures

• Target parameter for the primary hypothesis is subjective stress perception (PSS score)

Secondary Outcome Measures

• Scores of the questionnaires on resilience, quality of life, somatic symptoms, personality structure
• Dietary history: Food Frequency Items
• Patient satisfaction
• Subjective health literacy
• Blood parameters: differential blood count, CRP, IL-6, fasting blood glucose, HOMA index, cholesterol, HDL, LDL, triglycerides, ALT, AST, GGT, creatinine, urea, electrolytes, uric

acid, vitamin D, ferritin, homocysteine, vitamin B12
• Demographic data

Methods

Intervention

Participants receive a link to the questionnaires (via the EvaSys system) at baseline and subsequently at three-month intervals. They are asked to complete them within one week. Standard laboratory tests are also carried out at three-month intervals, but during normal follow-up appointments in the specialized clinic. There is no study-specific blood draw.

Questionnaires

The Perceived Stress Scale 10 (PSS10) is used to assess subjective stress burden.
The SF-12 is a questionnaire for measuring health-related quality of life. It is a shortened version of the SF-36 and includes questions on physical functions and limitations, pain perception, general health status, vitality, social and emotional limitations and psychological distress. The EQ-5D-5L is additionally collected to obtain a more comprehensive picture of quality of life.

Resilience is assessed using the Brief Resilience Scale (BRS-D). The Somatic Symptom Scale (SSS) provides information on experienced somatic symptoms.

To assess psychological structure, the short version of the OPD Structure Questionnaire (OPD-SFK) is used. The questionnaire is based on the manual of Operationalized Psychodynamic Diagnostics (OPD-2) and is used for self-assessment of central structural abilities, particularly in the areas of self- and other-perception, control ability, emotional regulation and relationship formation. The short version enables an economical yet valid assessment of structural levels of functioning in the clinical context with 12 items. The scales show good internal consistency as well as criterion-related validity in various patient populations.
A stable structural level is considered a central prerequisite for healthy stress processing, as it significantly influences the ability for self-regulation in stressful situations.

At baseline, a dietary history is taken using the NutriMental Screener to gain insight into eating habits and nutritional status.
Food Frequency Items are recorded both at baseline and at follow-ups to document dietary changes and compliance with dietary recommendations.

To evaluate patient satisfaction in the specialized clinic, questionnaires are used that have been adapted from those of other clinics at LKH Graz. These include, among other things, questions on satisfaction with organization/processes, professional competence/care, tone of interaction/behavior toward patients, as well as open questions on overall impression and suggestions for improvement.

Both patients and treating physicians are asked to assess the patients' health literacy on a scale from 1 (very low) to 10 (very high) at all assessment time points.

In addition, we collect the following clinical and demographic parameters: age, sex, height, BMI, medication, smoking behavior, chronic organic diseases, psychiatric diagnosis, educational status, employment/employment level.

Completion of the baseline questionnaires takes approximately 20 minutes, and completion of the follow-up questionnaires approximately 10–15 minutes.

Standard Laboratory

Blood samples will be taken at baseline and then every three months during the regular follow-up appointments by staff in the specialized outpatient clinics. A total of approx. 45 ml of blood (approx. 3 tablespoons) will be drawn and analyzed in a routine laboratory.

The following routine parameters are of particular interest for this study: differential blood count, CRP, IL-6, fasting blood glucose, HOMA index, cholesterol, HDL, LDL, triglycerides, ALT, AST, GGT, creatinine, urea, electrolytes, uric acid, vitamin D, ferritin, homocysteine, vitamin B12

Statistics

Data Analysis and Evaluation

The quantitative data collected in this study will be analyzed using IBM SPSS. First, a descriptive data description will be carried out, calculating mean, standard deviation and percentiles to present the basic characteristics of the sample and the measured variables.
To analyze changes in parameters over the course of treatment, both paired t-tests and repeated-measures analyses of variance (ANOVA) will be used. These procedures make it possible to assess differences between measurement time points (e.g., baseline vs. follow-up) and to reveal possible effects of the interventions. Prior to conducting these tests, normal distribution of the data will be examined, for example using the Kolmogorov–Smirnov test or the Shapiro–Wilk test.
In addition, relationships between variables will be examined using correlation analyses. Depending on data distribution, either the Pearson correlation coefficient (for normally distributed data) or the Spearman correlation coefficient (for non-normally distributed data) will be applied. Error probabilities of $p < 0.05$ will be considered statistically significant.
To investigate specific group comparisons and further detailed questions, post-hoc analyses may also be conducted.
The qualitative data from the patient satisfaction questionnaires will be evaluated by frequency analyses and a thematic analysis to identify common themes and key aspects of satisfaction. These complement the quantitative results and provide a more comprehensive insight into the patient perspective.

Considerations on Sample Size

As this is a pilot study, no exact sample size calculation can be given.
From January to August 2024, the specialized clinic recorded 91 initial consultations. Assuming that the number of new patients remains constant, we expect around 10 new patients per month, i.e., 120 per year. If 70% of these participate in the study, the number of study participants can be roughly estimated at around 84 people.

For the psychocardiology clinic, a regular frequency of four appointments per week over 52 calendar weeks is assumed, corresponding to a theoretical total capacity of 208 appointments per year. Taking into account public holidays, sick leave and vacation time, it is realistic to assume that about 75% of these appointments will be filled. This results in an estimated number of approximately 150 available appointment slots per year.
Since individual patients are seen multiple times, the actual number of individual new patients is lower than the total number of contacts. In comparable care settings, the average number of annual contacts per patient typically varies between 2.5 and 3 appointments. Based on this experience, a realistic estimate is around 50 to 60 new contacts per year.
In the liver clinic, an average of 5–6 patients are currently treated per Monday. The actual number varies depending on appointment scheduling, the presence of study patients and short-notice cancellations. Assuming approximately 46 active clinic days per year (taking into account public holidays, vacation and cancellations), the annual total number of patient contacts is approximately 230 to 276.
The number of new patients is estimated at 1 to a maximum of 2 per Monday, which—based on 46 actual clinic days—results in an approximate number of 46 to 92 first contacts per year.

Ethics and Data Protection

The study will be conducted in accordance with the ethical principles of the Declaration of Helsinki and national and international regulations. Participants will be comprehensively informed about the objectives, methods, potential benefits and risks of the study. Consent to participate is voluntary and must be given in writing. Participants' personal information will be treated confidentially and used only in anonymized form for data analysis.

Benefit–Risk Assessment

We assume that subjective stress perception can be reduced during care in the specialized outpatient clinics. Hardly any physical complaints and/or side effects are to be expected from completing questionnaires.

By precisely and repeatedly examining biopsychosocial variables (including stress, nutrition, resilience, quality of life), for which a debriefing of the results is also offered, participants also gain insight into possible personal treatment progress. They can then use this knowledge to engage more specifically with strategies to improve lifestyle.

Engaging with the psychological questionnaires may possibly trigger emotional reactions. However, the researchers will take care to prepare participants for such possibilities in advance and to offer supportive measures. In addition, completing questionnaires on a screen may lead to fatigue and headaches. Breaks can be taken at any time, and completion can be paused or discontinued. Routine blood draws may cause temporary discomfort, such as mild pain or temporary bruising at the puncture site. In rare cases, infections may occur. The researchers will ensure that experienced professionals perform these procedures to minimize the risk of complications.

The study will be conducted in accordance with the ethical principles of the Declaration of Helsinki and national and international regulations. Participants will be comprehensively informed about the objectives, methods, potential benefits and risks of the study. Consent to participate is voluntary and must be given in writing. Participants' personal information will be treated confidentially and used only in anonymized form for data analysis.

This pilot study can yield important insights into potential new treatment and prevention options in psychosomatics. Further studies can be based on this and, in the long term, new therapeutic and preventive options may emerge.
Participant feedback regarding their satisfaction in the clinic is of great importance for optimizing patient care.
The benefit–risk ratio is therefore positive.

Legal Aspects

The study will be conducted in accordance with applicable national and international legal provisions as well as ethical guidelines. All participants will be informed about their rights and obligations, and it will be ensured that their consent is voluntary and informed. Data protection regulations will be strictly observed to ensure the confidentiality of the data collected.

Publication and Dissemination of Results

The results of this study are to be published in renowned scientific journals in order to inform the scientific community about the findings. Authorship and publications will follow the guidelines of the International Committee of Medical Journal Editors (ICMJE). In addition, the results will be presented at scientific conferences to promote the exchange of knowledge and experience with other professionals.

Limitations and Challenges

This pilot study has some limitations and challenges that must be considered when interpreting the results.
First, the study duration may be too short to observe statistically significant effects or lasting changes, as the effects of nutritional and psychological interventions often take longer to fully develop. Second, the study is limited to a small number of participants in the specialized clinic, which may reduce statistical power and thus the likelihood of detecting significant results. This increases the risk of a Type II error, in which true effects are not recognized as significant due to insufficient power.
Another potential issue is the use of self-report measures (questionnaires), which are susceptible to biases such as social desirability or recall effects and can thus impair the reliability and validity of the data collected. In addition, compliance with specific therapeutic recommendations in the specialized clinics, such as dietary recommendations, is difficult to control, and individual differences in nutrient intake and utilization could influence the results of this study and lead to increased variability that occurs independently of the intervention.
Due to financial and logistical constraints, it is not possible within the scope of this study to conduct more specific nutritional diagnostic tests that would allow for even more precise tailoring of interventions. These limitations affect the significance and generalizability of the results but at the same time indicate important starting points for future studies. These could include a larger sample size, longer follow-up periods and a more differentiated nutritional diagnostic approach.

Summary

This pilot study aims to investigate the effectiveness of treatment and overall patient satisfaction in Europe’s first Specialized Outpatient Clinic for Nutrition and Mental Health as well as in other specialized clinics with innovative care concepts

(psychocardiology/psychohepatology). The study uses a single-center, prospective design and collects both subjective (questionnaires) and objective (blood values) parameters. The results of this study will help to improve patient care in a targeted manner and to collect initial data on the effectiveness of clinically hitherto little-tested nutrition-specific interventions in psychosomatics.

References

Berding, K., Bastiaanssen, T.F.S., Moloney, G.M., Boscaini, S., Strain, C.R., Anesi, A., Long-Smith, C., Mattivi, F., Stanton, C., Clarke, G., Dinan, T.G., Cryan, J.F., 2023. Feed your microbes to deal with stress: a psychobiotic diet impacts microbial stability and perceived stress in a healthy adult population. Mol Psychiatry 28, 601-610.

GBD 2019 Mental Disorders Collaborators. Global, regional, and national burden of 12 mental disorders in 204 countries and territories, 1990–2019: a systematic analysis from the Global Burden of Disease Study 2019. The Lancet Psychiatry. 2022 Jan;9(1):30-49. doi: 10.1016/S2215-0366(21)00395-3.

Jacka FN, O'Neil A, Opie R, Itsiopoulos C, Cotton S, Mohebbi M, et al. A randomized controlled trial of dietary improvement for adults with major depression (the “SMILES” trial). BMC Medicine. 2017;15(1):23. doi:10.1186/s12916-017-0791-y.

Maes, M., Yirmyia, R., Noraberg, J., Brene, S., Hibbeln, J., Perini, G., Kubera, M., Bob, P., Lerer, B., Maj, M.J.M.b.d., 2009. The inflammatory & neurodegenerative (I&ND) hypothesis of depression: leads for future research and new drug developments in depression. 24, 27-53.

Mörkl S, Wagner-Skacel J, Lahousen T, Lackner S, Holasek SJ, Bengesser SA, Painold A, Holl AK, Reininghaus E. The Role of Nutrition and the Gut-Brain Axis in Psychiatry: A Review of the Literature. Neuropsychobiology. 2018 Sep 17:1-9. doi: 10.1159/000492834. Epub ahead of print. PMID: 30223263.

Rush AJ, Trivedi MH, Wisniewski SR, et al. Acute and longer-term outcomes in depressed outpatients requiring one or several treatment steps: a STAR*D report. Am J Psychiatry. 2006;163(11):1905–1917. doi:10.1176/ajp.2006.163.11.1905.

Safadi, J.M., Quinton, A.M.G., Lennox, B.R., Burnet, P.W.J., Minichino, A., 2022. Gut dysbiosis in severe mental illness and chronic fatigue: a novel trans-diagnostic construct? A systematic review and meta-analysis. Molecular Psychiatry 27, 141-153.
